# MDMA-induced indifference to negative sounds is mediated by the 5-HT_2A_ receptor

**DOI:** 10.1007/s00213-017-4699-1

**Published:** 2017-07-22

**Authors:** K. P. C. Kuypers, R. de la Torre, M. Farre, N. Pizarro, L. Xicota, J. G. Ramaekers

**Affiliations:** 1 Department of Neuropsychology and Psychopharmacology, Faculty of Psychology and Neuroscience, Maastricht University, Maastricht, the Netherlands; 2 Integrative Pharmacology and Neurosciences Systems Research Group, Institut Hospital del Mar d’Investigacions Mèdiques, Barcelona, Spain; 3 Spanish Biomedical Research Centre in Physiopathology of Obesity and Nutrition (CIBEROBN), Santiago de Compostela, Spain; 4 CEXS-UPF, Universitat Pompeu Fabra, Barcelona, Spain; 5 Universitat Autonoma de Barcelona, Barcelona, Spain; 6 Clinical Pharmacology, Hospital Universitari Germans Trias i Pujol, Badalona, Spain

**Keywords:** MDMA, 5-HT_2A_ receptor, Ketanserin, Sound processing, Cognitive bias, Oxytocin, Arousal

## Abstract

**Background:**

MDMA has been shown to induce feelings of sociability, a positive emotional bias and enhanced empathy. While previous research has used only visual emotional stimuli, communication entails more than that single dimension and it is known that auditory information is also crucial in this process. In addition, it is, however, unclear what the neurobiological mechanism underlying these MDMA effects on social behaviour is. Previously, studies have shown that MDMA-induced emotional excitability and positive mood are linked to the action on the serotonin (5-HT) 2A receptor.

**Aim:**

The present study aimed at investigating the effect of MDMA on processing of sounds (Processing of Affective Sounds Task (PAST)) and cognitive biases (Approach-Avoidance Task (AAT)) towards emotional and social stimuli and the role of 5-HT_2A_ receptor in these effects.

**Methods:**

Twenty healthy recreational users entered a 2 × 2, placebo-controlled, within-subject study with ketanserin (40 mg) as pre-treatment and MDMA (75 mg) as treatment. Behavioural (PAST, AAT) measures were conducted 90 min after treatment with MDMA, respectively, 120 min after ketanserin. Self-report mood measures and oxytocin concentrations were taken at baseline and before and after behavioural tests.

**Results:**

Findings showed that MDMA reduced arousal elicited by negative sounds. This effect was counteracted by ketanserin pre-treatment, indicating involvement of the 5-HT_2_ receptor in this process. MDMA did not seem to induce a bias towards emotional and social stimuli. It increased positive and negative mood ratings and elevated oxytocin plasma concentrations. The reduction in arousal levels when listening to negative sounds was not related to the elevated subjective arousal.

**Conclusion:**

It is suggested that this decrease in arousal to negative stimuli reflects potentially a lowering of defences, a process that might play a role in the therapeutic process.

## Introduction

In recent years, a number of experimental studies have consistently shown that single doses of MDMA (e.g., 75–125 mg) cause an increase in emotional empathy (e.g., (Schmid et al. 2014; Hysek et al. 2014; Kuypers et al. 2014)). Empathy is only one aspect of social behaviour, and tests are in general based on visual stimuli, e.g., including the Reading the Mind in the Eyes test, the Facial Emotion Recognition Task and the Multifaceted Empathy Test. However, social interactions entail more than one sensory dimension, i.e., the auditory component also plays an important role in this process (Musson-Moyer 2012; Niedenthal 2007). Since all previous experimental MDMA studies have used visual stimuli, the effect of MDMA on the processing of auditory stimuli is not known. The present study therefore included a paradigm assessing the cognitive aspect of auditory stimulus recognition (‘which sound do you hear?’) and the emotional aspects linked to sound processing, i.e., sound-induced arousal and self-concern (‘how does this make you feel?’).

Previously, it has been shown that MDMA decreases amygdala activity and consequently might cause a reduction in avoidance behaviour (Gamma et al. 2000; Johansen and Krebs 2009; Bedi et al. 2009). In addition, it has been suggested that MDMA induces a positive emotional bias since participants rated favourite memories as significantly more vivid, emotionally intense and positive during MDMA intoxication and worst memories as less negative, compared to placebo (Carhart-Harris et al. 2014). The induction of such a cognitive bias and the reduction in avoidance behaviour by MDMA would explain why MDMA increases sociability and empathy. A task often used to study cognitive bias is by means of an approach-avoidance task. This paradigm is based on research indicating that flexions and extensions of the arm when using a joystick are, respectively, related to implicit positive evaluation (approach) and negative evaluation (avoidance) (Neumann and Fritz 2000).

Serotonin (5-HT) and oxytocin systems are known to be involved in approach-avoidance behaviour; i.e., it was shown previously that intranasal administered oxytocin regulates 5-HT_1A_ receptors and thereby interferes with 5-HT transmission, at the root of the 5-HT system, in the dorsal raphe nucleus (Cools et al. 2008). Domes and colleagues showed that intranasal application of oxytocin led to a valence-independent modulation of the approach bias and a reduction of amygdala activity to positive and negative stimuli (Domes et al. 2007). MDMA has previously been shown to induce an elevation in peripheral oxytocin concentrations (Kuypers et al. 2014; Dumont et al. 2009), and this might be a mechanism by which cognitive biases are induced by MDMA. In the present study, we were interested to test whether MDMA induces a bias towards emotional and social stimuli and whether this correlates with oxytocin concentrations.

Pre-clinical evidence showed that changes in the 5-HT_2A_ receptor responsivity after repeated administration of MDMA were accompanied by decreased social behaviour suggesting a role for this receptor in MDMA-induced sociality (Bull et al. 2004). Studies in humans have shown that the 5-HT_2A/C_ plays an important role in emotion and mood. Liechti and colleagues demonstrated that by blocking the 5-HT_2A_ receptor with ketanserin (50 mg), MDMA (1.5 mg/kg p.o.)-induced emotional excitation was attenuated (Liechti et al. 2000). In addition, van Wel and colleagues showed that the effects of a single dose of MDMA (75 mg) on positive mood were blocked by pre-treatment with ketanserin (50 mg) while this was not observed for negative mood effects (van Wel et al. 2012). Since mood and emotional excitability both play a role in the empathic response and social behaviour, and are linked with the activation of the 5-HT_2A_ receptor, it was hypothesised that blockade of this receptor could prevent or even counteract MDMA-induced behavioural responses to emotional stimuli.

The present study was set up to assess the effects of a single dose of MDMA (75 mg) on processing of emotional sounds, implicit attitudes towards social and emotional stimuli, mood, oxytocin responses and the role of the 5-HT_2A_ receptor herein.

## Materials and methods

### Participants

Participants were 20 healthy poly-drug MDMA users (12 males), with a mean (SD) age of 21.2 (2.6) years. They had previous experience with ecstasy/MDMA use and other drugs (Table 1). They were recruited through advertisements in university buildings and a website (digi-prik.nl) and by word of mouth.

**Table 1:** Self-reported lifetime history of drug use

Drug use (number of times used in lifetime)	Min	Max	Mean (SD)	*N* never used	Number
Ecstasy/MDMA	3	100	16.8 (23.2)	0	20
Amphetamine	1	3	2.2 (1.0)	16	4
Cannabis	1	300	54.2 (76.2)	2	18^a^
Cocaine	2	20	7.4 (6.4)	12	8
Mushrooms	1	50	8.6 (18.3)	13	7
LSD	20	20	20.0 (0.0)	19	1

^a^One participant answered: ‘I have used this so often, I cannot estimate how often I have used it’

### Design and treatments

The study was conducted according to a 2 × 2 double-blind, placebo-controlled, within-subject design including a pre-treatment and a treatment. Pre-treatment was a ketanserin capsule (40 mg), or placebo; treatment was an MDMA capsule (75 mg) or placebo. Capsules were administered orally with water, and all capsules were identically appearing. Ketanserin 40 mg represents a regular therapeutic dose that blocks about 91% of the 5-HT_2_ receptors (Brogden and Sorkin 1990; Sharpley et al. 1994).

A permit for obtaining, storing and administering MDMA was obtained from the Dutch drug enforcement administration. Randomization of pre-treatment and treatment conditions was generated by means of a Latin square, with each subject being assigned to a treatment sequence.

### Procedures

Prior to participation, all participants were medically assessed by a physician, who examined general health (including an ECG) and took blood and urine samples for standard chemistry and haematology. After study inclusion and before actual test days, participants were familiarized with the procedures, tests and questionnaires on a training day. They were requested to abstain from any drug use 1 week before the medical examination until the last test day. Participants were asked not to use any caffeinated or alcoholic beverages 24 h before testing and to get a normal night’s sleep as assessed.

Prior to experimental sessions, at 9 a.m., participants were screened for drugs of abuse consumption in urine (THC, opiates, cocaine, amphetamines, methamphetamines) and had to pass a breathalyser ethanol test. Women were given a pregnancy test. When tests were negative, participants had breakfast, filled out a questionnaire to assess their mood state (Profile of Mood States (POMS)) and a blood sample was taken to assess baseline oxytocin concentrations. Thereafter, pre-treatment was administered followed 30 min later by treatment. POMS and blood samples were taken 90 min after treatment and followed by behavioural tests. After the test battery, i.e., 150 min after treatment and 180 min after pre-treatment, POMS and blood samples were taken again and the test day ended. Test days were minimally separated by a 7-day wash-out period.

The study was performed in accordance with the Helsinki Declaration of 1975 and its amendments and was approved by the Medical Ethics Committee of the Academic Hospital of Maastricht and the University of Maastricht. Participants signed an informed consent and were paid upon completion of the testing periods for their participation.

### Processing of Affective Sounds Task

The Processing of Affective Sounds Task (PAST) was constructed to measure the effect of MDMA on processing affective sounds. Sixty sounds with each a duration of 6 s (30 positive/pleasant, 30 negative/unpleasant) were selected from the International Affective Digital Sounds database of (Bradley and Lang 2000) which contains a set of 111 standardized, emotionally evocative sounds that cover a wide range of semantic categories (Stevenson and James 2008). Previously, in a study of Stevenson and James (Stevenson and James 2008), participants classified to which extent these sounds belonged to one of five emotional categories (happy, sad, fear, disgust, anger) and how aroused the sounds made them feel. For the purpose of our study, we selected the 30 most positive/pleasant (happy) and 30 most negative/unpleasant (sad (4), fear (19), anger (1), disgust (6)) non-verbal sounds. Norm data from the selected sounds showed that on a nine-point scale, the ‘positive’ sounds were judged as significantly more pleasant (average rating ± SE [7.1 ± 0.9]) than negative sounds (2.8 ± 1.4) (*t*_29_ = 16.1; *p* < 0.001) and negative sounds were experienced as more arousing (6.5 ± 0.2) compared to positive sounds (4.8 ± 0.2) (*t*_29_ = −6.7; *p* < 0.001).

Sounds were presented subsequently through headphones. Participants had to type in what they thought they heard (cognitive component, recognition) and subsequently had to categorize the sound as pleasant (rating from one to three), neutral (zero) or unpleasant (rating from −1 to −3) and rate how aroused the perception of this sound made them (scale from one to nine) (emotional component). Only ‘arousal’ and ‘feeling’ ratings from correctly identified sounds were averaged to yield a mean Arousal or Feeling rating.

### Implicit attitude tests

Three versions of the Approach-Avoidance Task (AAT) were used to test automatic action tendency towards presented stimuli (pictures). These included AATs to measure approach-avoidance behaviour to social, threat and trust stimuli. Participants were instructed to push or pull a joystick as fast as possible upon appearance of the stimuli, according to the rules, independently of the content of the stimulus. They had to push when the pictures were tilted to the right respectively to the left and pull when the pictures were tilted 3% to the left respectively to the right. Upon movement of the joystick, the picture changed in size such that it grew upon pulling and shrank upon pushing, creating the visual impression that the picture itself is being pulled closer (approach) or pushed away (avoidance). After participants moved the joystick and the picture changed format, the picture disappeared, irrespective of whether it was a correct response. Each image was presented four times, i.e., twice in pull and twice in push format. The resulting trials were presented in a semi-random order, i.e., at most three similar rotations and image categories in a row and preceded by 15 practice trials with grey rectangles (procedure cfr. (Cousijn et al. 2011)). The dependent variable was a bias score which was calculated by subtracting median approach reaction time (RT) from median avoid RT for each image category. The bias could either be positive (approach bias) or be negative (avoidance bias) indicating the automatic action tendency towards or away from a scene (Social AAT) or an emotion (Threat AAT/Trust). Before this bias was calculated, data were corrected for outliers meaning that extreme high (>2000 ms or ≥3 SD above the individual participants’ mean RT) or extreme low reaction times (<200 ms or ≤3 SD from the individual participants’ mean RT) were removed (procedure cf. (Cousijn et al. 2011)).

For both the Threat AAT and the Trust AAT, 24 pictures (12 neutral + 12 high threat; 12 neutral + 12 high trustworthy) were selected from the data set of Oosterhof and Todorov (Oosterhof and Todorov 2008). This resulted for both tasks in 96 trials. For the Social AAT, 36 black and white pictures were selected containing either nature scenes (neutral category) or scenes with one (‘social low’) or multiple persons (‘social high’). Twelve pictures per category resulted in 144 trials.

### The Profile of Mood States

The POMS (de Wit et al. 2002) is a self-assessment mood questionnaire with 72 five-point Likert scale items, representing eight mood states; i.e., Anxiety, Depression, Anger, Vigour, Fatigue, Confusion, Friendliness and Elation. Two extra scales are derived, i.e., Arousal ((Anxiety + Vigour) − (Fatigue + Confusion)) and Positive mood (Elation − Depression). The participant had to indicate to what extent these items were representing his/her mood.

### Pharmacokinetics and oxytocin concentrations

Blood samples were collected three times on each test day in order to determine oxytocin concentrations and pharmacokinetics of MDMA, MDA and ketanserin.

### Pharmacokinetics

Samples were centrifuged immediately, and resulting plasma was stored at −20 °C until analysis. MDMA were determined by gas chromatography coupled to mass spectrometry using a method previously described by Pizarro and colleagues (Pizarro et al. 2002). Ketanserin was determined by liquid chromatography coupled to mass spectrometry. Samples (200 μL of plasma) were purified with Ostro Pass-through Sample Preparation Plates (Waters, MA, USA) and 600 μL of acetonitrile with 0.1% formic acid were used as the elution solvent. After mixing, vacuum was applied and the collected mixture was evaporated to dryness at 15 psi and 40 °C. Extract was reconstituted with 100 μL of ammonium formate 0.02% at pH 5 and acetonitrile (50:50 *v*/*v*). Quantification was performed in an HPLC system coupled to a triple-quadrupole (6410 Triple Quad LC-MS; Agilent) mass spectrometer with an electrospray interface. The chromatographic separation was done using a C18 column (Kinetex, 100 mm × 3 mm × 1.7 μm, Phenomenex, CA, USA). The mobile phase was ammonium formate 0.02% at pH 5 and acetonitrile in an isocratic mode (50:50 *v*/*v*) at a flow rate of 0.45 mL/min. All compounds were monitored in positive ionization using the multiple reaction mode mass/charge (*M* + 1/*z*) values selected for identification of analytes were as follows: ketanserin 396 → 146, 189, 208; and pirenperone 394 → 119, 159, 187, fragmentor (F) 200 V, collision energy (CE).

### Oxytocin concentrations

A 2-mL sample for oxytocin analysis was drawn and collected in non-heparinized tubes. Samples were centrifuged at 3500 rpm for 10 min and at 4 °C. Serum was removed and frozen at −80 °C until analysis. Serum oxytocin concentrations were determined by a fluorescent immunoassay kit (Phoenix Pharm. Inc., Burlingame, CA) following the manufacturers’ instructions, briefly a first extraction step using C18 columns was performed, followed by a fluorimetric EIA analysis as per protocol.

### Statistical analyses

Data entered a general linear model (GLM) repeated measures analysis of variance (RM ANOVA) procedure (SPSS, version 24.0) with Pre-treatment (two levels: ketanserin, placebo) and Treatment (two levels: MDMA, placebo) as main within-subject factors. Extra within-subject factors were included for the PAST (Valence, two levels) and the AATs (Stimulus category, two levels (Trust, Threat), or three levels (Social)). In case of interaction effects, paired *t* tests were conducted to test the origin of the interaction.

For the POMS and oxytocin concentrations, three measures were collected including baseline. First, a GLM RM ANOVA was conducted, including only baseline to test for baseline differences. In case there were no differences, another GLM was conducted, including only the second or third measure to test for main and interaction effects of Pre-treatment and Treatment.

To study whether ketanserin and MDMA plasma concentrations differed significantly between conditions in which ketanserin or MDMA was administered alone or in combination, paired sample *t* tests were conducted.

The alpha criterion level of statistical significance for all analyses was set at *p* = 0.05; effect sizes are reported as partial eta-squared (*ƞ*_p_^2^) (magnitude: small = 0.01, moderate = 0.06, large = 0.14).

## Results

### Processing of Affective Sounds Task

**Fig. 1:**
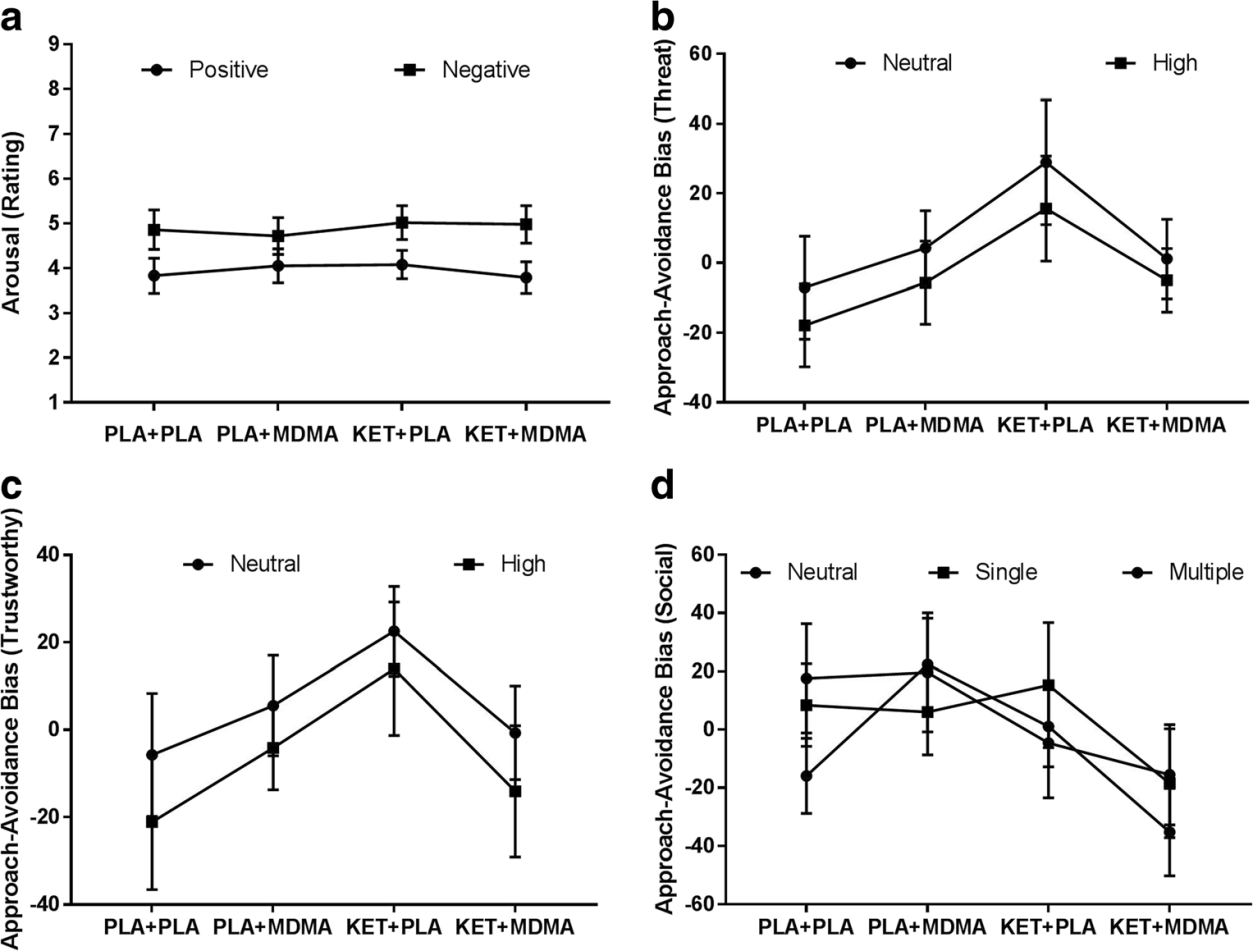
Mean (±SE) arousal rating of positive and negative sounds in the Processing of Affective Sounds Task in (**a**), and mean (±SE) approach-avoidance biases of threat stimuli (**b**), trustworthy stimuli (**c**) and social stimuli (**d**)

Analyses revealed a main effect of Valence on ratings of Feeling (*F*_1, 19_ = 154.31; *p* < 0.001, *ƞ*_p_^2^ = 0.89) and Arousal (*F*_1, 19_ = 22.30; *p* < 0.001, *ƞ*_p_^2^ = 0.54). Positive sounds were rated as more positive compared to negative sounds, and negative sounds produced more arousal compared to positive sounds. Furthermore, there was a three-way interaction of Pre-treatment × Treatment × Valence (*F*_1, 19_ = 8.80; *p* = 0.008, *ƞ*_p_^2^ = 0.32) on Arousal. When under influence of MDMA alone, participants were equally aroused by positive and negative sounds in contrast to the placebo (*t*_19_ = −2.59; *p* = 0.02) and the combined ketanserin-MDMA condition (*t*_19_ = −2.66; *p* = 0.02) where participants were more aroused by negative stimuli. This behavioural pattern in the MDMA only condition could not be attributed to an increase in rating of the positive sounds compared to placebo as shown by insignificant ad hoc *t* test (*t*_29_ = 1.3; *p* = 0.2). When MDMA was combined with ketanserin, the MDMA-induced ‘indifference’ with regard to the valence of the sound disappeared and a differentiation between arousal ratings for positive and negative sounds was observed, i.e., arousal for negative sounds was higher than arousal experienced when hearing positive sounds. This pattern resembled placebo ratings (Fig. 1a).

Otherwise, no main effect of Pre-treatment, Treatment or their interaction on dependent variables of the PAST was found (Table 2).

**Table 2:** Mean (±SE) and GLM RM main effects of Valence (V), Pretreatment (PT) and Treatment (T) on Feeling and Arousal during negative (−) and positive (+) sounds in the Processing Auditory Stimuli Task (PAST); *p* = – depicts statistical insignificance at *p* < 0.05

	Mean (±SE) ratings				Main effects RM GLM ANOVA					
PT	PLA	PLA	KET	KET	V		PT		T	
T	PLA	MDMA	PLA	MDMA	*F*_1, 19_	*p*	*F*_1, 19_	*p*	*F*_1, 19_	*p*
Feeling +	1.0 (0.1)	1.2 (0.1)	1.1 (0.1)	1.3 (0.1)	154.31	<0.001	0.34	–	1.49	–
Feeling −	−0.9 (0.1)	−0.8 (0.1)	−0.9 (0.1)	−0.9 (0.1)						
Arousal +	3.8 (0.4)	4.0 (0.4)	4.1 (0.3)	3.8 (0.3)	22.30	<0.001	0.21	–	0.43	–
Arousal −	4.9 (0.4)	4.7 (0.4)	5.0 (0.4)	5.0 (0.4)						

### Implicit attitude tests

#### Threat AAT

Analyses revealed no main effect of Pre-treatment or Treatment on Threat Bias. The interaction between Pre-treatment and Treatment approached significance (*F*_1, 19_ = 4.13, *p* = 0.056, *ƞ*_p_^2^ = 0.18). Data suggested that treatment with ketanserin alone increased the approach tendency towards faces, irrespective of the emotional expression compared to placebo; whereas the combination of ketanserin with MDMA or MDMA alone resulted in an avoidance bias that was close to zero (Fig. 1b). There was no main effect of Stimulus category on Threat Bias, i.e., while Threat neutral faces were approached (6.81) and Threat-High faces were avoided (−3.25), biases did not statistically significant differ.

#### Trust AAT

Analysis revealed a Pre-treatment by Treatment interaction (*F*_1, 19_ = 5.17, *p* = 0.03, *ƞ*_p_^2^ = 0.21); additional analyses showed that this interaction was due to significant differences between the ketanserin and the placebo condition (*t*_19_ = 2.85; *p* = 0.01) and ketanserin and the combined condition (*t*_19_ = 2.21; *p* = 0.04). During placebo, participants displayed an avoidance bias (−13.5), independent of (emotional) stimulus content; ketanserin alone led to an approach bias. Under influence of MDMA, the (approach) bias was close to zero (i.e., 0.7). When ketanserin was combined with MDMA, there was an avoidance bias (−7.4), which was comparable to placebo (−13.46). It therefore seems that when combined with MDMA, the ketanserin’s approaching effects are eliminated and even reversed. Analyses revealed no main effect of Pre-treatment or Treatment. There was no main effect of the Stimulus category, i.e., while Trustworthiness-neutral faces were approached (bias 5.4) and Trustworthiness-high faces were avoided (bias −6.4), they did not statistically significant differ (Fig. 1c).

#### Social AAT

Analyses revealed a main effect of Pre-treatment on Social bias (*F*_1, 19_ = 4.62, *p* = 0.04, *ƞ*_p_^2^ = 0.20); i.e., under influence of ketanserin, participants had an avoidance bias (−9.6), irrespective of stimulus content, compared to the placebo conditions (9.7). The Pre-treatment by Treatment interaction approached significance (*F*_1, 19_ = 4.16, *p* = 0.056, *ƞ*_p_^2^ = 0.18) suggesting that when ketanserin (approach bias 3.9) and MDMA (approach bias 16) were combined, this led to an avoidance bias (−23.1; *t*_19_ = 2.4; *p* = 0.03), indicating a negative synergy. There was no main effect of the Stimulus category; the bias for pictures with single and multiple people (approach biases 2.8 and 4.3) did not differ significantly from the bias for natural scenes (avoidance bias −6.9) (Fig. 1d).

### Profile of Mood States

There were no baseline differences in POMS scores over the four test days. Analysis including the second (pre-test) measurement revealed main effects of Pre-treatment and Treatment on 7 out of 10 scales of the POMS. The effects of ketanserin and MDMA were in opposing directions on Vigour, Elation, Arousal, Positive Mood and Fatigue. While ketanserin increased fatigue, MDMA caused a decrease; in contrast, ketanserin decreased vigour, elation, arousal and positive mood, while MDMA increases these subjective feelings. Effects on Anxiety and Confusion were the same for ketanserin and MDMA; both seemed to increase ratings. Analysis including the third (post-test) measurement yielded approximately the same results with the exception of one difference and four additional effects. The effect of ketanserin on Anxiety was in the opposite direction compared to the second measurement, i.e., ketanserin caused a decrease in Anxiety. During the third measurement, there were two additional main effects of Pre-treatment and Treatment on Friendliness; i.e., ketanserin reduced friendliness, while MDMA increased friendliness. Three additional interaction effects were revealed on Anxiety, Elation and Arousal. When ketanserin was combined with MDMA, effects of MDMA were counteracted and ratings were placebo like, i.e., anxiety, elation and arousal were reduced compared to the MDMA only condition (Table 3).

**Table 3:** Mean (±SE) and GLM outcomes of dependent variables of the POMS; 1 = POMS baseline measurement, 2 = POMS measurement before cognitive tests (peak drug) and 3 = POMS measurement after cognitive tests (end of test day); *p* = – depicts statistical insignificance at *p* < 0.05

Mood scales	Mean (±SE) mood scales					Main and interaction effect GLM RM ANOVA					
	PT	PLA	PLA	KET	KET	PT		T		PT × T	
	T	PLA	MDMA	PLA	MDMA	*F*_1, 19_	*p*	*F*_1, 19_	*p*	*F*_1, 19_	*p*
Anxiety	1	2.7 (0.3)	2.8 (0.3)	2.5 (0.3)	3.0 (0.5)	0.94	–	0.36	–	0.50	–
	2	2.0 (0.3)	4.8 (0.9)	2.4 (0.3)	4.7 (0.6)	0.08	0.00	32.42	0.00	0.21	–
	3	2.5 (0.2)	5.8 (0.7)	3.0 (0.3)	3.2 (0.4)	9.73	0.00	18.62	0.00	13.73	0.001
Depression	1	0.1 (0.1)	0.2 (0.1)	0.2 (0.2)	0.4 (0.3)	0.65	–	0.43	–	0.07	–
	2	0.2 (0.2)	0.1 (0.1)	0.4 (0.2)	0.6 (0.3)	2.58	–	0.00	–	0.61	–
	3	0.4 (0.1)	0.5 (0.2)	0.5 (0.2)	0.2 (0.1)	0.15	–	0.18	–	1.75	–
Anger	1	0.7 (0.2)	0.8 (0.2)	1.2 (0.3)	0.9 (0.3)	2.65	–	0.12	–	0.56	–
	2	0.8 (0.3)	0.9 (0.2)	0.5 (0.2)	0.8 (0.3)	0.96	–	2.49	–	0.80	–
	3	0.9 (0.3)	1.2 (0.3)	0.6 (0.0)	0.4 (0.2)	8.89	–	0.18	–	1.77	–
Vigour	1	11.2 (1.1)	12.2 (1.2)	11.9 (1.3)	11.7 (1.1)	0.04	–	0.41	–	0.80	–
	2	10.0 (1.2)	13.7 (1.3)	6.5 (1.1)	11.4 (1.7)	9.09	0.001	16.72	0.001	0.41	–
	3	9.4 (1.2)	13.8 (1.4)	6.10 (0.8)	9.0 (1.4)	34.10	0.00	18.54	0.00	2.37	–
Fatigue	1	1.3 (0.5)	1.4 (0.4)	1.4 (0.4)	1.9 (0.6)	0.85	–	0.64	–	0.29	–
	2	2.1 (0.6)	1.0 (0.3)	6.7 (1.4)	4.5 (1.5)	10.69	0.03	5.73	0.03	0.44	–
	3	4.3 (1.3)	1.7 (1.2)	7.4 (1.5)	5.3 (1.2)	18.27	0.04	4.79	0.04	0.10	–
Confusion	1	3.1 (0.2)	3.6 (0.4)	3.3 (0.3)	3.4 (0.6)	0.01	–	0.89	–	0.36	–
	2	3.7 (0.33)	4.7 (0.5)	5.1 (0.5)	7.2 (0.9)	9.51	0.01	7.43	0.01	1.92	–
	3	4.7 (0.6)	6.4 (0.6)	6.1 (0.4)	7.6 (0.9)	17.51	0.03	5.87	0.03	0.05	–
Friendliness	1	16.8 (1.2)	17.5 (1.4)	17.3 (1.6)	18.0 (1.2)	0.56	–	0.82	–	0.00	–
	2	15.9 (1.4)	16.7 (1.8)	14.6 (1.4)	16.6 (1.5)	0.66	–	2.93	–	0.73	–
	3	15.2 (1.4)	18.4 (1.5)	13.4 (1.6)	15.9 (1.5)	7.66	0.006	9.58	0.006	0.18	–
Elation	1	10.4 (0.8)	11.2 (0.8)	10.9 (1.0)	11.5 (0.8)	0.86	–	2.02	–	0.04	–
	2	10.0 (1.0)	12.6 (1.1)	8.4 (0.7)	11.3 (1.3)	4.19	0.005	9.92	0.005	0.06	–
	3	8.6 (0.9)	13.3 (1.0)	7.4 (1.0)	10.2 (1.0)	12.26	0.000	25.28	0.00	4.87	0.04
Arousal	1	9.5 (1.5)	10.0 (1.4)	9.6 (1.6)	9.4 (1.6)	0.05	–	0.02	–	0.13	–
	2	6.1 (1.5)	12.8 (1.8)	−2.8 (2.2)	4.3 (3.5)	9.30	0.001	14.30	0.001	0.01	–
	3	3.0 (2.3)	11.5 (2.5)	−4.4 (1.9)	−0.8 (2.6)	39.07	0.004	10.98	0.004	6.49	0.02
Positive mood	1	10.3 (0.9)	11.0 (0.9)	10.7 (1.0)	11.1 (1.0)	0.28	–	0.79	–	0.07	–
	2	9.7 (1.0)	12.5 (1.1)	8.0 (0.8)	10.7 (1.4)	4.49	0.007	8.97	0.007	0.00	–
	3	8.2 (1.0)	12.8 (1.0)	6.9 (1.0)	9.9 (1.0)	7.55	0.00	23.91	0.00	3.37	–

### Drug pharmacokinetics and oxytocin concentrations

#### Pharmacokinetics

Paired sample *t* tests showed that MDMA plasma concentrations (ng/mL) did not statistically differ between the MDMA alone condition (mean (±SE): 90′ post-MDMA: 134.8 (16.6); 150′ post-MDMA: 186.0 (17.7)) and the condition where it was combined with ketanserin (mean (±SE): 90′ post-MDMA: 126.7 (15.1); 150′ post-MDMA: 182.9 (14.7)). The same was shown for ketanserin plasma concentrations (ng/mL), i.e., they did not differ between the ketanserin alone condition (mean (±SE): 90′ post-MDMA: 54.9 (7.6); 150′ post-MDMA: 64.5 (6.0)) and the condition where it was combined with MDMA (mean (±SE): 90′ post-MDMA: 59.0 (8.8); 150′ post-MDMA: 61.5 (5.4)).

#### Oxytocin concentrations

Analyses revealed no baseline differences in oxytocin serum concentrations between treatment conditions. There was a main effect of MDMA (*F*_1, 13_ = 5.71; *p* = 0.03, *ƞ*_p_^2^ = 0.30) on oxytocin concentrations 90 min after administration, i.e., MDMA caused an increase in oxytocin concentrations (0.8 ± 0.1 pg/mL) compared to placebo (0.5 ± 0.1 pg/mL). There was no main effect of Pre-treatment or a Pre-treatment by Treatment interaction on oxytocin concentrations, 90′ after MDMA treatment.

Analyses revealed a main effect of Pre-treatment (*F*_1, 11_ = 16.90; *p* = 0.002, *ƞ*_p_^2^ = 0.61) and a Pre-treatment by Treatment interaction effect (*F*_1, 11_ = 5.29; *p* = 0.04, *ƞ*_p_^2^ = 0.32) on oxytocin concentrations, 150′ after MDMA administration. The main effect demonstrated a decrease in oxytocin concentrations after ketanserin treatment (0.3 ± 0.1 pg/mL) compared to placebo (0.8 ± 0.2 pg/mL). The interaction effect showed that ketanserin blocked the MDMA-induced elevation in oxytocin concentrations.

### Post hoc correlations

Seen the effects of MDMA on Arousal in the PAST, correlations between this parameter and Arousal and Anxiety of the POMS and oxytocin concentrations were calculated in order to explore the relation between general levels of anxiety and arousal, oxytocin levels and task-related arousal. The correlations (*r*_20_) were not statistically significant and ranged between −0.2 and 0.2 for POMS arousal-PAST arousal, −0.1 and 0.3 for POMS anxiety-PAST arousal and −0.3 and 0.3 for oxytocin concentrations-PAST arousal.

In addition, since oxytocin is involved in social behaviour and ketanserin blocked the effects of MDMA on oxytocin concentrations, and three POMS scales, i.e., Anxiety, Elation and Arousal, correlations were calculated to test whether POMS and oxytocin concentrations were associated. Pearson’s correlations were not statistically significant for the three scales, i.e., *r*_20_ = 0.3 for POMS anxiety, *r*_20_ = 0.4 for POMS arousal and *r*_20_ = −0.1 for POMS elation.

## Discussion

The aim of the present study was to investigate the effect of MDMA (75 mg) on processing of auditory stimuli; implicit attitudes towards social and emotional stimuli, mood and oxytocin concentrations and the role of the 5-HT_2A_ receptor in these effects. It was demonstrated that MDMA reduced arousal experienced by negative sounds. This effect was counteracted by ketanserin. Findings showed in general no effects of MDMA on approach-avoidance behaviour. Ketanserin, however, induced avoidance behaviour in the Social AAT, independent of stimulus content, and approach behaviour in the Trust AAT, when it was administered alone. When combined with MDMA, approach behaviour in the Trust AAT was decreased. MDMA caused, in line with previous findings (Kuypers et al. 2014; van Wel et al. 2012), an increase in positive and negative mood, it lowered ratings of fatigue and it increased oxytocin plasma concentrations. Ketanserin counteracted a selection of MDMA-induced mood effects 150 min after MDMA intake.

In the Processing of Affective Sounds task, it was shown that MDMA reduced arousal induced by negative sounds; pre-treatment with ketanserin changed the MDMA response, i.e., increasing the arousal experienced by negative sounds and thereby returning the arousal levels to a placebo-like response. This placebo response was in line with the norm data on which the task was based (Stevenson and James 2008) and previous findings in non-drug using volunteers, showing that listening to unpleasant sounds induced larger startle reflexes and larger heart rate decelerations compared with listening to pleasant sounds. It was suggested that these sounds activated the appetitive and defensive motivational circuits (Bradley and Lang 2000). Our findings suggest an MDMA-induced indifference to negative sounds, or an inhibition of fear induced by negative sounds, which could hint at an inhibition of in-built fear responses (Phillips et al. 1998). These findings are also in line with a rodent study, showing disruption of pre-pulse inhibition after treatment with MDMA, which was counteracted by a selective 5-HT_2A_ receptor antagonist. It was concluded that the effect of MDMA was caused by action on this receptor (Padich et al. 1996).

The absence of significant correlations between self-rated arousal levels on the POMS in the separate drug conditions and the arousal elicited by the emotional sounds demonstrates that the observed effects were not simply related to a general MDMA effect on arousal levels but were stimulus dependent. While the general level of arousal was rated higher in the MDMA condition and lower when MDMA was combined with ketanserin, this pattern was opposite when confronted with auditory stimuli in the PAST. So, during MDMA intoxication participants experienced more general arousal but stimulus-related arousal was less, especially when confronted with negative stimuli. This is a very interesting reaction, and it can be suggested that participants felt less discomfort or more at ease when they heard negative sounds. This could contribute to the therapeutic potential of MDMA in a therapeutic setting, i.e., when patients are asked to talk about their negative experiences and then being confronted in an auditory way with the negative wording, their arousal levels might, not increase, their defences might be lowered which has been suggested to contribute to the therapeutic process (Greer and Tolbert 1990).

MDMA induced in general no cognitive bias, as suggested by the absence of effects on the approach-avoidance tasks. In the Trust-AAT, it counterintuitively decreased the ketanserin-induced stimulus-intensity-independent approach behaviour. Interestingly, many studies have shown that approach and avoidance arm movements are facilitated by positive and negative affect, respectively (Phaf et al. 2014). MDMA is known to cause an increase in both affective states (van Wel et al. 2012), e.g., increasing positive mood but in parallel also elevating feelings of anxiety and confusion. This effect on both mood states could explain the lack of hypothesised effects. Separately blocking the positive and negative mood effects of MDMA could be used to test whether MDMA induces a mood-congruent cognitive bias, i.e., approaching when in a positive mood and avoiding when in a negative mood state. Previously, it was shown that negative mood effects (anxiety) could be blocked by using verbal support during an MDMA session (Vizeli and Liechti 2017), while positive effects were inhibited by using ketanserin in combination with MDMA (van Wel et al. 2012). In addition, while previous research demonstrated that intranasal administration of oxytocin modulates approach bias (Domes et al. 2007), it was shown that the MDMA-induced increase in oxytocin plasma concentrations is smaller than the increase induced by intranasal administered oxytocin (Kuypers et al. 2014). The latter potentially also explains why in the present study, no MDMA-induced bias was found.

In line with previous studies (Kuypers et al. 2014; Dumont et al. 2009), MDMA caused an increase in oxytocin concentrations, 90 and 150 min after administration, i.e., oxytocin plasma concentrations were doubled compared to placebo. Interestingly, ketanserin blocked this MDMA-induced elevation in oxytocin concentrations but only 150 min after MDMA administration, i.e., 180 min after pre-treatment with ketanserin. This effect was not observed 90 min after MDMA administration, respectively, 110 min after ketanserin pre-treatment. The ketanserin-induced changes in oxytocin concentrations were not associated with the ketanserin-induced changes in MDMA-mood effects. Ketanserin works as an antagonist on 5-HT_2A_ and 5-HT_2C_ receptors, and previously, animal studies have shown that the 5-HT_2C_ receptor is involved in oxytocin secretion (Jørgensen et al. 2003). It is therefore suggested that the MDMA-induced elevation of oxytocin concentrations could be attributed to its action on the 5-HT_2C_ receptor, though this needs to be investigated further. In addition to this, previous research has shown the oxytocin receptor gene to be associated with sociability feelings during MDMA intoxication (Bershad et al. 2016) and with the efficiency of social auditory processing (Tops et al. 2011). Together, these findings suggest that the oxytocin receptor gene in combination with actions on the 5-HT_2C_ receptor could also mediate the efficiency of sound processing after MDMA administration.

In conclusion, present findings seem to suggest that a single dose of MDMA (75 mg) does not induce a specific bias towards emotional or social stimuli but it reduces arousal experienced by listening to negative sounds. The latter effect seems to be mediated by the 5-HT_2A_ receptor, since ketanserin blocked this effect. This behavioural effect was not related to subjectively experienced arousal. It is suggested that this seemingly lowering of defences when confronted with negative stimuli might play a role in the therapeutic process.
